# How the Loss of Second Molars Corresponds with the Presence of Adjacent Third Molars in Chinese Adults: A Retrospective Study

**DOI:** 10.3390/jcm11237194

**Published:** 2022-12-03

**Authors:** Li-Juan Sun, Yang Yang, Zhi-Bang Li, Yi Tian, Hong-Lei Qu, Ying An, Bei-Min Tian, Fa-Ming Chen

**Affiliations:** 1 State Key Laboratory of Military Stomatology, National Clinical Research Center for Oral Diseases, Department of Periodontology, School of Stomatology, Fourth Military Medical University, 145th West Changle Road, Xi’an 710032, China; 2 Department of Oral and Maxillofacial Surgery, The 940th Hospital of Joint Logistics Support Force of PLA, Lanzhou 730050, China

**Keywords:** second molar, tooth loss, tooth extraction, third molar

## Abstract

Third molars (M3s) can increase the pathological risks of neighboring second molars (M2s). However, whether the M3 presence affects M2 loss remains unknown. This retrospective study aimed to reveal the reasons for M2 loss and how M2 loss relates to neighboring M3s. The medical records and radiographic images of patients with removed M2(s) were reviewed to analyze why the teeth were extracted and if those reasons were related to adjacent M3s. Ultimately, 800 patients with 908 removed M2s were included. In the included quadrants, 526 quadrants with M3s were termed the M3 (+) group, and the other 382 quadrants without M3s were termed the M3 (−) group. The average age of patients in the M3 (+) group was 52.4 ± 14.8 years and that of the M3 (−) group was 56.7 ± 14.9 years, and the difference between the two groups was statistically significant (*p* < 0.001). Of the 908 M2s, 433 (47.7%) were removed due to caries and sequelae and 300 (33.0%) were removed due to periodontal diseases. Meanwhile, 14.4% of the M2s with adjacent M3s were removed due to distal caries and periodontitis, which were closely related to the neighboring M3s; this percentage was much lower when M3 were absent (1.8%). Additionally, 42.2% of M3s were removed simultaneously with neighboring M2s. The presence of M3s, regardless of impaction status, was associated with an earlier loss of their neighboring M2s.

## 1. Introduction

Tooth loss is a global problem that not only impacts the daily lives of patients but is also associated with various systemic diseases [1,2,3]. Clinical evidence suggests that, in addition to the third molar (M3), the second molar (M2) is one of the most frequently removed permanent teeth in adults [4,5]. Similar to other teeth, many factors may affect an M2′s life span, such as oral hygiene, occlusal condition, trauma and smoking [6,7,8,9]. Among the various risk factors for M2 pathology, the presence of neighboring M3s has attracted increasing attention [10,11,12,13]. In this context, studies have confirmed that the presence of impacted M3s (I-M3s) significantly increases the risk of M2 pathology [11,12]. In recent years, investigators have found that nonimpacted M3s (N-M3s) also negatively impact oral health [11,12,14]. When M2 damage is too severe to be treated or controlled, M2s can only be removed.

To prevent M2 loss, we should try to identify the early pathologies (i.e., caries and periodontitis) before irreversible damage occurs, and minimizing the risk factors that cause M2 caries and periodontitis is equally important. With regard to well-known risk factors, the presence of M3s, preventive M3 extractions may be the most comprehensive solution. Substantial evidence shows that the incidence rates of caries and periodontal pathologies can be decreased following M3 extraction [15,16,17,18]. However, the decision to preventatively extract asymptomatic M3s is challenging for dentists as well as patients. In many clinical situations, asymptomatic M3s are more likely to be retained until irreversible damage occurs to their neighbors [19,20]. Unfortunately, researchers have found that even though the health status of M2s is actively monitored, there is still a significant rate of M2 mortality [21,22].

Whether the loss of M2s corresponds with the presence of adjacent M3s remains unclear. Evaluating the reasons for M2 loss in clinics may help dentists better understand the relationship between M2 loss and their neighboring M3s, which will allow them to perform timely interventions to actively protect M2s from M3-related damage. This study aimed to identify the reasons why M2s were extracted and their relationship with the presence of their neighboring M3s.

## 2. Materials and Methods

### 2.1. Inclusion and Exclusion Criteria

This was a retrospective study based on the medical records and radiographic images of patients who had their M2(s) removed in the School of Stomatology, Fourth Military Medical University (FMMU), Xi’an, China. The clinical material of patients who had visited the Department of Oral and Maxillofacial Surgery from January 2019 to March 2019 was continuously screened. The inclusion criteria were as follows: (i) the patients were ≥18 years old; (ii) the patients had at least one M2 extracted; and (iii) the patients had complete and explicit medical records and radiographic images (obtained by orthopantomograms) of their extracted M2s (prior to extraction) and their neighboring M3s (if any). Patients with incomplete medical records or inadequate radiographic image qualities for either the extracted M2 or neighboring M3 were excluded from this study. In addition, patients with discrepant medical records and radiographic images were excluded from the analysis.

### 2.2. Reasons for M2 Extraction

The reasons for M2 extraction were mainly classified into the following three categories: (i) severe caries and sequelae, -whenever the primary indication for extraction was caries or its sequelae, e.g., untreatable caries, residual roots, pulp and periapical tissue diseases; (ii) severe periodontitis, -when the M2 was extracted for periodontal breakdown; and (iii) others, -orthodontic needs, cysts or tumors, noncarious defects (e.g., tooth fracture or root resorption), and other ambiguous reasons for M2 extraction. When periodontal disease and caries (or its sequelae) presented in the M2 at the same time, only the predominant reason for M2 extraction was recorded. In this case, if it was not possible to identify which disease was more likely the main cause of M2 loss, it was recorded as a comorbidity and assigned to the category of “others”.

To study whether M2 loss was related to their adjacent M3s, the M2s were further divided into the following categories according to the location of diseases in M2s: mainly occurred on the distal surface of M2s, mainly occurred on other surfaces except for the distal surface, and impossible to confirm whether the disease occurred on the distal surface.

### 2.3. Data Collection

The patients’ demographic information (age, gender, systematic diseases, smoking status, etc.) and details about the extracted M2s (prior to extraction) and their neighboring M3s (disease and clinical situation of the M2s before extraction, the status of M3s, etc.) were continuously collected from the electronic medical system (doctors) of the hospital. To minimize errors, the radiographic images were observed in a digital viewer (Hi Net, Hwatech, Xi’an, China) to judge whether findings from the radiographic images were consistent with the medical record data (especially the reasons for M2 extraction). All the data collections were conducted by two trained independent investigators (L.S. and Y.Y.), and the intraexaminer reproducibility was 95%.

### 2.4. Groups

Based on the absence/presence and status of M3s, the quadrants with the extracted M2s were divided into the M3 (−) group (quadrants with extracted M2s and without M3s) and M3 (+) (quadrants with extracted M2s and with M3s). In this context, quadrants in the M3 (+) group were further divided into the N-M3 group (quadrants with M2s extracted with nonimpacted M3s that erupted to the occlusal plane) and the I-M3 group (quadrants with extracted M2s and impacted M3s that unerupted to the occlusal plane), wherein quadrants with extracted M2s that had neighboring M3s that were residual roots or microdontia were excluded from analysis.

### 2.5. Statistical Methods

EpiData (version 3.0, EpiData Association, Odense, Denmark) was used to collect data, and SPSS (version 20, IBM, Chicago, IL, USA) was used to analyze the data. Missing or abnormal data were checked again and refilled according to the patients’ medical records and radiographic images. The normality of qualitative data (age) was tested by the Shapiro–Wilk test. The results showed that the age of all groups was nonnormally distributed. Therefore, the differences in age between the two groups were tested by Mann–Whitney U tests. The differences in quantitative data (gender, jaw, right/left and indications) between the two groups were tested by chi-square tests. The two-sided significance level was set at *p* < 0.05.

## 3. Results

### 3.1. Clinical Material for Analysis

**Figure 1:**
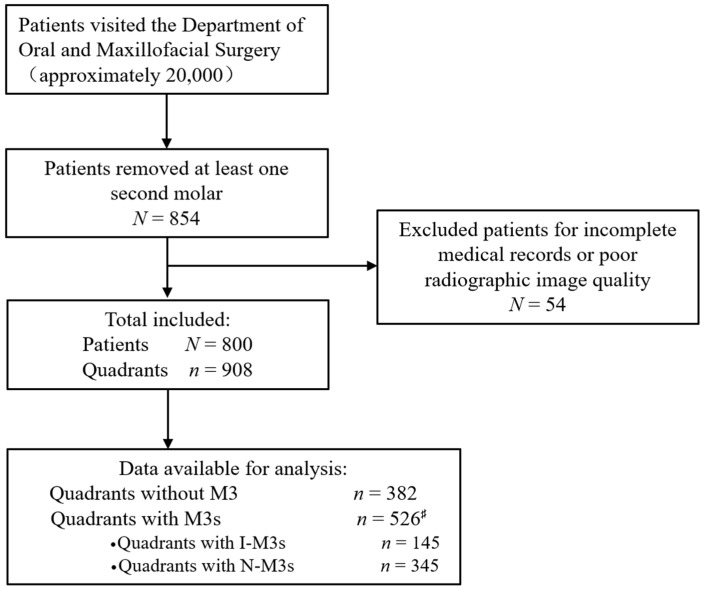
Flow chart of the study design. ^♯^: including 145 quadrants with I-M3s, 345 quadrants with N-M3s and 36 quadrants with M3s that were residual roots or microdontia.

According to the electronic medical records system, approximately twenty thousand people visited the Department of Oral and Maxillofacial Surgery from January to March 2019. Among them, 854 patients had at least one M2 removed. The clinical material of 54 patients was excluded from this study due to incomplete medical records, poor radiographic image quality or both reasons. Finally, the clinical material of 800 patients was utilized for analysis in this study (Figure 1).

### 3.2. Characteristics of the Patients

The characteristics of the patients whose clinical material was utilized for investigation are shown in Table 1. Most of the subjects (87.6%) had only one M2 removed. The age of these enrolled patients ranged from 19 years to 91 years, and the mean age was 54.1 (standard deviation, SD: 15.0) years. Of all the subjects, 95.3% were nonsmokers. The proportion of males (54.6%) was higher than that of females (45.4%). In addition, the prevalence of systematic disease(s) among these patients was 36.0%, and among the patients with systematic diseases, 36.1% suffered a combination of several systematic diseases.

### 3.3. Characteristics of the Quadrants with Extracted M2s

Clinical material of the 800 patients (908 quadrants with 908 extracted M2s) was applied for analysis at the quadrant (tooth) level. In general, M2 extraction was more likely to occur between the ages 50 and 59, and two-thirds of M2s were removed between the ages of 40 and 69 years. Compared to females (44.6%), more male patients (55.4%) had their M2s extracted. Slightly over half of the extractions were undertaken in the mandibular (51.7%) region. There was no difference in M2 extraction that occurred on the left side (49.8%) and right side (50.2%). According to the patients’ medical records and radiographic images, only 209 of the 908 (23.0%) M2s had been treated for diseases (caries, periodontal disease, prosthesis, etc.) before M2 extraction.

According to the absence or presence of adjacent M3s, the 908 quadrants with M2s that were removed were divided into the M3 (−) group and the M3 (+) group; 382 quadrants were within the M3 (−) group, and 526 quadrants were within the M3 (+) group. Table 2 shows the age and distribution of the extracted M2s adjacent to the different statuses of M3s. The average age of patients in the M3 (−) group was 56.7 (SD: 14.9) years, while in the M3 (+) group, it was 52.4 (SD: 14.8) years. When the normality of age was tested by the Shapiro–Wilk test, it was found that the ages of the M3 (−) group and M3 (+) group were nonnormally distributed. The presence of M3s corresponded with an earlier loss of their neighboring M2s (Mann–Whitney U test, *p* < 0.001). The distributions of quadrants with M2s that were extracted were also different according to the absence/presence and different statuses of M3s neighboring the extracted M2s. In the M3 (+) group, the proportion of male patients was 59.5%, which was higher than that in the M3 (−) group (49.7%); the difference was statistically significant (*p* = 0.003). In addition, in the M3 (+) group, there were more quadrants with M2s extracted in the mandible (56.8%) than in the maxillary region, while in the M3 (−) group, there were more quadrants with M2s extracted in the maxillary region (*p* < 0.001). In addition, regarding the distribution of quadrants with M2s extracted on the left/right side, no significant difference was found between the two groups (*p* = 0.229).

Except for the 36 quadrants in which the M3s were residual roots or microdontia, the other 490 quadrants in the M3 (+) group were further divided into the N-M3 group (quadrants with M2s extracted with nonimpacted M3s) and the I-M3 group (quadrants with M2s extracted with impacted M3s). The numbers of the two groups were 145 and 345, respectively. The mean age of patients in the N-M3 group was 54.2 (SD: 14.3) years and that of the I-M3 group was 46.2 (SD: 14.4) years (Table 2). Patients who had extracted M2s that neighbored I-M3s were younger than those who had extracted M2s that neighbored N-M3s (*p* < 0.001). In addition, the distribution of quadrants with M2s extracted in the maxillary/mandibular region was different according to the different statuses of the M3s. A total of 82.1% of extracted M2s in the mandible occurred in the I-M3 group, which was much higher than that in the N-M3 group (*p* < 0.001). There was no significant difference between the I-M3 group and the N-M3 group regarding gender or side (right/left) (*p* = 0.312 and *p* = 0.559, respectively).

### 3.4. Reasons for M2 Extraction

When the reasons for M2 extraction were examined (Figure 1), nearly half of the M2s (47.7%) were extracted due to caries, and the consequences of M2 caries; among them, 61.7% of the M2s were residual roots. Another 33.0% of M2s were removed due to local advanced periodontitis, and the remaining 19.3% of extractions were due to other reasons, including tooth fracture, comorbidities, cysts and tumors. It was interesting that in the category of “others”, noncarious tooth fracture was the most common reason, and approximately 1/10 of the 908 M2s were extracted for this reason.

**Figure 2:**
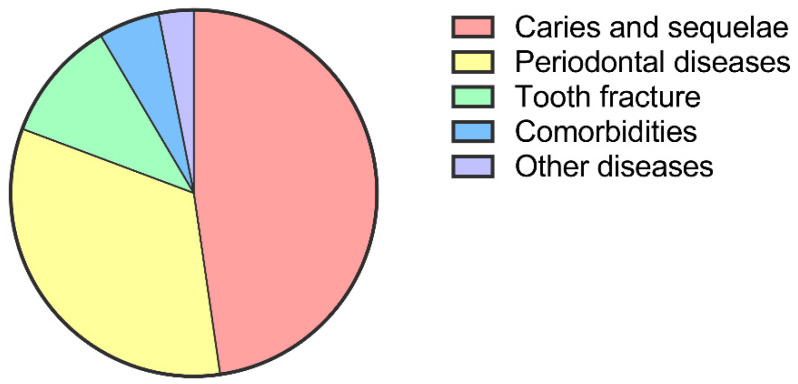
Reasons for M2 extraction in 908 quadrants.

The main reasons for extraction of the 908 M2s were caries and their sequelae (47.7%) and periodontal diseases (33.0%) (Figure 2). However, the distribution of these reasons (caries and sequelae, periodontal diseases and others) was not the same between M2s with/without neighboring M3s. Table 3 shows the reasons for M2 removal in these groups. Similar to the entire sample, the most common reason for M2 extraction in the M3 (+) group and the M3 (−) group was still caries and their sequelae. However, the distribution of indications for M2 removal between the two groups was different (*p* = 0.003). The percentage of M2 loss due to periodontal diseases was higher in the M3 (+) group than in the M3 (−) group. In addition, the prevalence of extracted M2s ascribed to periodontal diseases was higher in the N-M3 group (42.3%) than in the I-M3 group (29.7%, *p* = 0.009).

Then, whether M2 loss corresponded with their adjacent M3s was analyzed. As shown in Table 3, in the M3 (−) group, only 1.8% of M2s were extracted for diseases that mainly occurred on the distal surface; however, this proportion was much higher in the M3 (+) group (14.4%). In the I-M3 group, 64 M2s (44.1%) were extracted due to distal diseases, including 29 extractions caused by periodontal diseases, 24 extracted M2s due to caries and their sequelae, and 11 M2s lost to root resorption, cysts or other diseases. In the N-M3 group, although the rate of M2 extraction due to distal diseases (3.5%) was lower than that in the I-M3 group, it was still higher than that of M2s without neighboring M3s.

### 3.5. The Rate and Reasons for M3s Extracted with Neighboring M2s

Among the 908 quadrants, 526 had neighboring M3s next to the removed M2s, wherein 7 had been removed 1–4 years prior to M2s extraction. Among the 519 retained M3s (I-M3s: 143; N-M3s: 340; residual roots: 36), 237 were extracted along with the M2s; the other 282 M3s were retained at least before their clinical material was used in this study (Table 4). Further analysis indicated that approximately 2/3 (64.3%) of the I-M3s (92/143) were removed along with M2 extraction, while only 1/3 (32.9%) of the N-M3s (112/340) and their adjacent M2s were simultaneously extracted.

**Figure 3:**
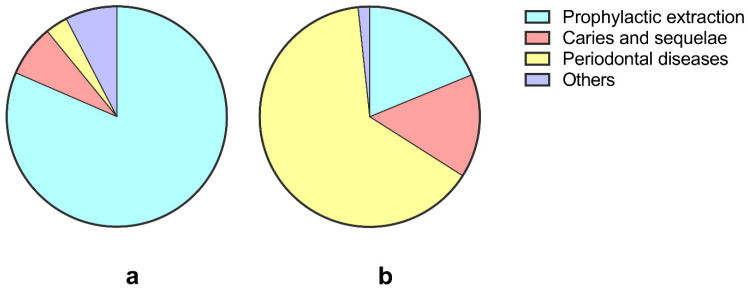
Reasons for extraction of I-M3s (**a**) and N-M3s (**b**) along with adjacent M2 extraction.

The reasons for M3 removal along with adjacent M2 extraction are further analyzed in Figure 3; indications for extraction were generally classified into the following categories: prophylactic extraction, caries and sequelae, periodontal diseases and others. Except for the 33 residual roots, 92 I-M3s and 112 N-M3s (Table 4) were enrolled in this analysis. The results showed that the distribution of M3 removal was very different between the I-M3 and N-M3 group (*p* < 0.001). Most I-M3s (81.5%) were disease-free or in the early stages of disease, and they were proactively removed during neighboring M2 extraction surgeries; only 7.6% of I-M3s were removed due to caries and sequelae, and 3.3% of I-M3s were extracted because of periodontitis (Figure 3a). However, across the extracted N-M3s, 4/5 were removed due to irreversible diseases (Figure 3b), wherein 64.3% (72/112) of N-M3s were extracted for periodontal diseases and 15.2% of N-M3s (17/112) were removed due to caries and sequelae. Among them, 18.8% of the N-M3s (21) were extracted prophylactically, and evidence showed that at least 12 of the 112 N-M3s were nonfunctional teeth.

## 4. Discussion

Unfortunately, there were many M2 extractions in the clinic. The authors analyzed 908 M2s extracted from 800 patients to reveal which individuals had M2s removed, why the M2s were removed, the degree to which M2 loss was related to their neighboring M3s and how the M3s were handled. The results showed that patients with M3s, regardless of I-M3s or N-M3s, may lose their neighboring M2s earlier than those without M3s; the prevalence of M2 loss due to caries and/or periodontal diseases on the distal surface (which were closely related to their neighboring M3s) was much higher in the quadrants with M3s than in those without M3s; nearly 2/3 I-M3s and 1/3 N-M3s were also removed during the neighboring M2 extraction surgeries.

Tooth loss can not only affect the daily quality of life of patients but is also associated with a variety of systemic diseases, including patient longevity [1,2]. Therefore, clarifying the patterns of tooth loss may help dentists protect teeth more efficiently. Caries and periodontal diseases are the two major reasons for tooth loss that were reported in previous studies [23,24,25], and they were consistent with the main reasons for M2 loss in this study. In addition, what makes M2s different from other teeth is the presence of neighboring M3s, which is the most frequently impacted tooth [26]. M3-related diseases include pericoronitis, caries, cysts, tumors and other forms of destruction adjacent to M2s [27,28,29]. The influence of M3s on adjacent M2s is widely considered. Studies have confirmed that the presence of M3s is a risk factor for adjacent M2s [11,12,14]. Thus, it is not impractical to separate M2s from neighboring M3s when examining the patterns of M2 loss.

In this study, the main reason for M2 extraction was caries and their sequelae, followed by periodontal diseases; however, the indicators were distributed differently in different groups. In the I-M3 group, approximately one-half of M2s were extracted for caries and their sequelae, and one-third of M2s were removed due to periodontal diseases. However, when M3s erupted, the percentage of periodontal diseases was much higher and almost reached the percentage of caries and their sequelae. In recent years, several studies have explored the negative effect of erupted M3s and found that the risk of M2s being destroyed by periodontal disease may increase 1.44–6.79-fold when erupted M3s are present [11,12,30,31]. In this study, the high percentage of periodontal diseases in the N-M3 group also allowed clinicians to focus not only on caries but also on periodontal diseases.

There are a large number of factors that affect the life spans of M2s [6,32], which is why to date, there is no direct evidence revealing the relationship of M2 loss with their neighboring M3s. It is generally believed that the negative effect of M3s mainly occurs in distal M2s [12,17,30]; thus, the prevalence of M2 loss due to diseases on the distal surface may reflect the influence of M3s on adjacent M2s. On the basis of this assumption, we compared the incidence of distal diseases in extracted M2s that were adjacent to M3s with different statuses. In this study, when M2s were divided into different groups according to the status of neighboring M3s, the findings were interesting. The results showed that the prevalence of M2 loss due to diseases on the distal surface was higher among M2s with adjacent M3s (14.4%) than among those without M3s (1.8%), especially when the M3s were impacted (44.1%). Although the rate of M2 extraction due to distal diseases (3.5%) was lower than that of the I-M3 group, the passive effect of erupted M3s on their neighboring M2s needs further examination. In a 25-year cohort study, scientists reported that during the follow-up, 14.6% to 39.1% of M2s adjacent to impacted M3s and 3.8% of M2s that neighbored erupted M3s were removed, while none of the M2s without M3s were extracted [21]. Combining the two studies, the authors concluded that the presence of M3s may lead to the loss of M2s. In addition, in this study, the diseases in four-fifths M2s were too severe to confirm where they mainly occurred, which means that diseases in some of these M2s may have originated from the distal surface. Moreover, most caries or periodontal diseases that occur only in distal M2s can be treated; thus, our study may underestimate the negative effect of M3s.

Although the presence of M3s is generally believed to increase the residual periodontal pockets and risk of caries in M2s [27,29,33], the decision for preventive extraction of asymptomatic M3s is difficult for dentists and patients [20,34]. In most studies, the preventive extraction of M3s can be beneficial for the health of neighboring M2s [15,18,35,36]; in many clinical situations, asymptomatic M3s are more likely to be retained until irreversible damage occurs [19,20]. Evidence of this was also found in this study. Nearly two-thirds of I-M3s and one-third of N-M3s were removed during the neighboring M2 extraction surgeries, and among them, 81.5% I-M3s and only 18.8% N-M3s were prophylactically extracted even when their M2s were heavily damaged. For patients who have low compliances and high risks, early intervention, such as prophylactical extraction of M3s, may be more beneficial for the health of adjacent M2s than waiting to act until severe diseases occur. It is time for a change.

Preventive extraction of M3s may be the most thorough way to protect M2s. However, M3 extraction is neither risk-free nor cost-free, especially for N-M3s that still have occlusal functions in the mouth. Therefore, the prophylactic removal of all asymptomatic M3s is unreasonable, and it is important to screen out high-risk patients and M2s. Studies have found that factors such as male gender and old age can increase the risk of M2 destruction [14,31,37]. Although the characteristics of the population included in this study could not verify the risk factors for M2s, the mean age of the patients was greater, and the percentage of males was higher than the percentage of females.

Many studies have confirmed that age is significantly associated with tooth loss [3,4]. In our study, the patients’ mean age was 54.1 years, which was similar to that of the studies that involved all types of permanent tooth loss [5,38]. Compared with M2s without adjacent M3s, the mean age at M2 extraction was younger when M3s were present. Especially for M2s with neighboring impacted M3s, the average age at M2 removal was greater by a whole decade. The authors speculate that the presence of M3s accelerated disease progression in neighboring M2s, and this hypothesis can be explained by other studies. Compared with M2s without neighboring M3s, retained impacted M3s have been confirmed to increase the risk of M2 pathology by 2.16- to 4.88-fold [11]. Interestingly, Kaye and colleagues recently reported that retained M3s were not associated with an increased risk of M2 loss among adult men (average age: 48 years) [39]. Their study excluded participants whose M2s were already extracted, and this leads us to the opposite conclusion.

Due to the large number of patients who were receiving outpatient care, it was difficult to examine every patient who met the criteria at the same time; moreover, the electronic record system was relatively complete and followed the unified standards, so we conducted a retrospective study. In addition, imaging data were collected to ensure the accuracy of the data. It was concluded that the presence of M3s may necessitate a sooner M2 extraction. Long-term retention of impacted M3s and erupted M3s may cause great harm to adjacent teeth.

This study had some limitations. It was difficult to judge whether the missing M3s were congenitally absent or whether those M3s had been removed before this investigation. If so, the influence of M3s would be underestimated. Second, when an M2 has severe periodontal disease, it easily falls out, so the actual percentage of M2 loss due to periodontal diseases may be higher than the reported figure in this study. Additionally, the data were collected from medical records that were filled out by other clinicians, which might lead to differences in the actual data recorded, and some data were not collected (e.g., oral hygiene, severity of systemic diseases), which could also lead to potential biases in the data analyses. Long-term prospective studies are needed to observe the effect of M3s on the health of adjacent teeth. In addition, investigations that target the general population rather than patients can come to a more reliable conclusion. Previous studies found that the presence of M3s can increase the risk of pathology in adjacent M2s [28,29]. In this study, the mean age and reasons for M2 extraction were different in the three groups, which only indicates that M3s may be related to M2 loss, but we still do not know the scale or degree of that influence. The results remind clinicians to pay more attention to the negative influence of M3s, regardless of their impaction or eruption, and to conduct timely clinical interventions before irreversible damage emerges in neighboring M2s.

## 5. Conclusions

M3-associated caries and periodontitis were two main reasons for the loss of adjacent M2s, and the presence of M3s was associated with an earlier loss of neighboring M2s. We conclude that the presence of M3s, regardless of I-M3s or N-M3s, may accelerate the loss of adjacent M2s. However, further high-quality studies based on a large population across various regions are needed to justify the accuracy of this finding.

## Figures and Tables

**Table 1:** Characteristics of the patients whose clinical material was utilized for analysis (*N* = 800).

Characteristics		*N*	%
Age (years)	Mean ± SD	54.1 ± 15.0	
	Median (Q25, Q75)	55.0 (44.0, 64.8)	
Gender	Male	437	54.6
	Female	363	45.4
Systematic disease(s)	No	512	64.0
	Yes	288	36.0
Smoker	No	762	95.3
	Yes	38	4.8
Distribution of extracted M2s among patients	1	701	87.6
	2	91	11.4
	3	7	0.9
	4	1	0.1

SD: standard deviation; Q25: upper quartile; Q75: lower quartile.

**Table 2:** Characteristics of the quadrants with extracted M2s (*n* = 908) †.

Characteristics		M3 (−) Group	M3 (+) Group						
			Total	*p*	I-M3 Group	*p*	N-M3 Group	*p*	*p* *
*n*		382	526 ^♯^		145		345		
Age (mean ± SD) (years)		56.7 ± 14.9	52.4 ± 14.8	<0.001	46.2 ± 14.4	<0.001	54.2 ± 14.3	0.024	<0.001
Gender (%)	Male	49.7	59.5	0.003	56.6	0.162	61.4	0.002	0.312
	Female	50.3	40.5		43.3		38.6		
Jaw (%)	Maxillary	55.5	43.2	<0.001	17.9	<0.001	51.6	0.292	<0.001
	Mandibular	44.5	56.8		82.1		48.4		
Right/Left (%)	Right	48.4	52.5	0.229	54.5	0.215	51.6	0.394	0.559
	Left	51.6	47.5		45.5		48.4		

†: grouped by the absence/presence and status (impacted/nonimipacted) of M3s neighboring the extracted M2s; ^♯^: including 145 quadrants with I-M3s, 345 quadrants with N-M3s and 36 quadrants with M3s that were residual roots or microdontia; *p*: compared with the M3 (−) group; *p* *: the N-M3 group compared with the I-M3 group.

**Table 3:** Reasons for M2 extraction in quadrants with the absence or presence (impacted/erupted) of neighboring M3s (*n* = 908).

Groups		*n*	Indication (%)				Location of Main Disease in M2s (*n*)		
			Caries and Sequelae	Periodontal Diseases	Others	*p*	Distal (%)	Nondistal	Uncertain
M3 (−)		382	49.0	27.7	23.3	Reference	7 (1.8)	5	370
M3 (+)	Total	526 ^♯^	46.8	36.9	16.3	0.003	76 (14.4)	19	431
	I-M3	145	46.9	29.7	23.4	0.892	64 (44.1)	6	75
	N-M3	345	43.2	42.3	14.5	<0.001	12 (3.5)	13	320
Total		908	47.7	33.0	19.3	-	83 (9.1)	24	801

^♯^: including 145 quadrants with I-M3s, 345 quadrants with N-M3s and 36 quadrants with M3s that were residual roots or microdontia. Others: orthodontic needs, cysts or tumors, noncarious defects (e.g., tooth fracture or root resorption), and other ambiguous reasons for M2 extraction.

**Table 4:** The rate of M3 removal in M2 extraction surgery (*n* = 519).

M3 (+) Group	*n*	Neighboring M3s Removed along with M2s			
		Yes (*n*)	%	No (*n*)	%
Total	519	237	45.7	282	54.3
I-M3	143	92	64.3	51	35.7
N-M3	340	112	32.9	228	67.1
Residual roots	36	33	91.7	3	8.3

## Data Availability

The data presented in this study are available on request from the corresponding author upon reasonable request.
